# Impact of Orthologous Gene Replacement on the Circuitry Governing Pilus Gene Transcription in Streptococci

**DOI:** 10.1371/journal.pone.0003450

**Published:** 2008-10-20

**Authors:** Sergio Lizano, Feng Luo, Farah K. Tengra, Debra E. Bessen

**Affiliations:** Department of Microbiology and Immunology, New York Medical College, Valhalla, New York, United States of America; Tufts University, United States of America

## Abstract

**Background:**

The evolutionary history of several genes of the bacterial pathogen *Streptococcus pyogenes* strongly suggests an origin in another species, acquired via replacement of the counterpart gene (ortholog) following a recombination event. An example of orthologous gene replacement is provided by the *nra/rofA* locus, which encodes a key regulator of pilus gene transcription. Of biological importance is the previous finding that the presence of the *nra*- and *rofA*-lineage alleles, which are ∼35% divergent, correlates strongly with genetic markers for streptococcal infection at different tissue sites in the human host (skin, throat).

**Methodology/Principal Findings:**

In this report, the impact of orthologous gene replacement targeting the *nra/rofA* locus is experimentally addressed. Replacement of the native *nra*-lineage allele with a *rofA*-lineage allele, plus their respective upstream regions, preserved the polarity of Nra effects on pilus gene transcription (i.e., activation) in the skin strain Alab49. Increased pilus gene transcription in the *rofA* chimera correlated with a higher rate of bacterial growth at the skin. The transcriptional regulator MsmR, which represses *nra* and pilus gene transcription in the Alab49 parent strain, has a slight activating effect on pilus gene expression in the *rofA* chimera construct.

**Conclusions/Significance:**

Data show that exchange of orthologous forms of a regulatory gene is stable and robust, and pathogenicity is preserved. Yet, new phenotypes may also be introduced by altering the circuitry within a complex transcriptional regulatory network. It is proposed that orthologous gene replacement via interspecies exchange is an important mechanism in the evolution of highly recombining bacteria such as *S. pyogenes*.

## Introduction

The human pathogen *Streptococcus pyogenes* (group A streptococci; GAS) has a past history of extensive genetic recombination [1]–[4]. Striking among these genetic changes is the substitution of several genes by an orthologous form originating from another bacterial species [5], [6]. Phylogenetic support for orthologous gene replacements in GAS lies in the occurrence of alleles at a given locus comprising ≥2 discrete lineages, whereby within-lineage sequence divergence is much lower than the between-lineage divergence. Orthologous genes are presumed to share many functions, yet are sufficiently divergent in sequence to confer novel phenotypes.

Orthologous gene replacements in *S. pyogenes* have been documented for loci encoding transcription regulatory proteins (*mga*, *nra/rofA*) and the extracellular virulence factor streptokinase (*ska*) [5], [6]. A presumptive donor species – the commensal-like *S. dygalactiae* subsp. *equisimilis* – is a likely source for divergent *nra/rofA* and *ska* genes. The GAS alleles found at each of these loci comprise two (*mga1/mga2*, *nra/rofA*) or three (*ska1/ska2a/ska2b*) discrete lineages. Furthermore, the *mga*, *nra/rofA* and *ska* loci occupy distant map positions on the GAS genome and are not physically linked. That orthologous gene replacement at these loci has biological significance is supported by the distribution of the lineage-specific alleles within the GAS population, whereby the *mga*, *nra/rof* and *ska* lineages each display strong linkage with a genetic marker for tissue site preference for infection at the throat or skin [5], [6]; the observed linkage disequilibrium occurs against a background of highly random associations between housekeeping genes [2].

The epithelium of the throat and skin of the human host constitutes the primary ecological niche for GAS. Genetic markers for tissue site preferences for infection lie within the *emm* region (denoted *emm* pattern), and are used to define throat specialist (*emm* pattern A–C), skin specialist (*emm* pattern D) and generalist (*emm* pattern E) strains, with each group having a predilection for causing infection at their respective tissue sites [7]. The correlation between *emm* pattern group and streptococcal disease at superficial tissue sites – pharyngitis and impetigo – finds strong support in numerous population-based surveillance studies (reviewed in [8]). The *mga* locus, comprised of two orthologous allelic forms, lies adjacent to the *emm* region.

The physically distant *nra/rofA* locus encodes a stand-alone transcriptional response regulator of FCT-region genes, encoding the proteins needed for biosynthesis of surface pili [9]–[13]. Nra and RofA have been characterized as both activators and repressors, depending on the GAS strain [14], [15]. The *nra*-lineage alleles of the *nra/rofA* locus show a statistically significant association with the *emm* pattern D skin specialist strains, whereas the *rofA*-lineage alleles are largely confined to the pattern A–C and E strains of throat specialists and generalists, respectively [5]. Therefore, is it reasonable to postulate that orthologous gene replacement at the *nra/rofA* locus was a pivotal step in establishing tissue site preferences for infection.

Precisely how the skin specialist phenotype emerged from a throat specialist or generalist phenotype, or vice versa, is difficult to know because phylogenetic relationships can be masked by the high levels of recombination characteristic of this species. The goal of this study is to experimentally reconstruct a genotype that represents a plausible intermediate step in the evolution of the tissue-specific infection phenotypes displayed by modern-day GAS. To this end, the *nra* allele of a pattern D skin specialist strain was replaced with a *rofA* allele, and its impact on transcriptional regulatory circuits and biological behavior was assessed.

## Results

### Replacement of *nra* with *rofA* preserves pilus gene transcription

**Figure 1:**
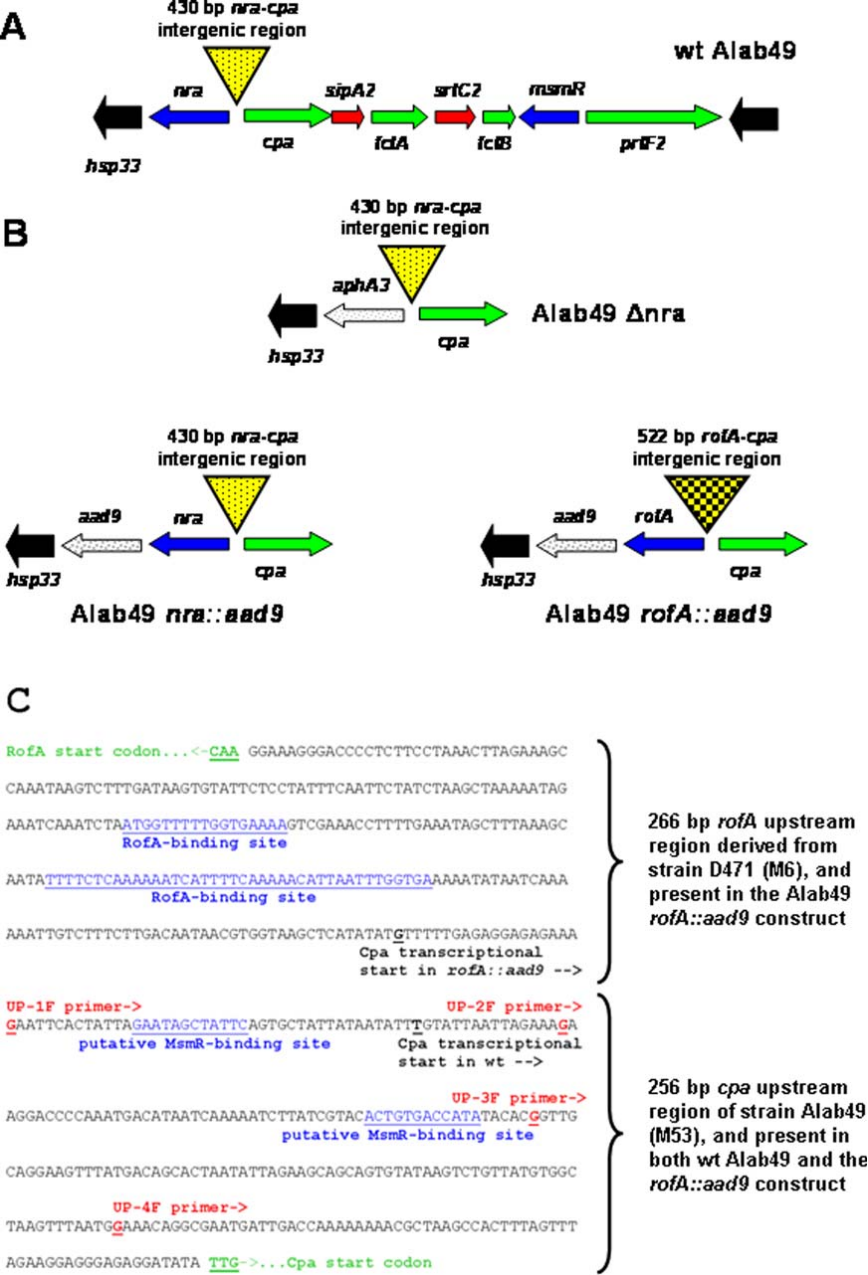
FCT-region maps of wt Alab49 and isogenic mutants. A, Map of the FCT-region of wt Alab49; the direction of transcription for each gene is shown (arrows). The four genes encoding surface proteins are in green; also shown are genes whose products have a role in transcriptional regulation (blue) and pilus assembly (red). The 430 bp intergenic region between *nra* and *cpa* is indicated by an inverted triangle. Black arrows represent the highly conserved genes that form the boundary of the FCT-region; the *hsp33*gene lies downstream of *nra*, outside of the FCT-region. B, Maps of the modified portions of the FCT-region for the Alab49 Δnra mutant and *nra::aad9* and *rofA::aad9* constructs. The *aad9* gene encoding spectinomycin resistance is inserted in the *nra-hsp33* intergenic region, at a position of 97 nt downstream of *nra/rofA* and 33 nt upstream of *hsp33*. C, Nucleotide sequence of the 522 bp *rofA-cpa* intergenic region within the Alab49 *rofA::aad9* chimeric construct, consisting of a 266 bp region upstream of *rofA* derived from strain D471 (M6), juxtaposed to the 256 bp region upstream of *cpa* derived from strain Alab49 (M53). Known and putative sequence motifs involved in DNA binding by RofA and MsmR (blue, underlined) are highlighted [12], [16], [22]. Also indicated are 5′ end positions for forward oligonucleotide primers (UP-1F through UP-4F; red, underlined) used in PCR amplification. The transcriptional start sites for *cpa* transcripts in both wt Alab49 and the *rofA::aad9* construct, as determined by RACE (see text), are depicted (black, underlined).

The first objective is to experimentally reconstruct a genotype that could plausibly represent an intermediate form in the evolutionary history of *S. pyogenes* [5], [10]. The *nra* gene of the classic skin strain Alab49 (M protein type 53, M53; FCT-3 region form) was chromosomally replaced with the *rofA* gene from the classic throat strain D471 (M protein type 6, M6; FCT-1 region form), using the Δnra mutant as a recipient (Figure 1A–B). The 1,536 bp *nra* gene, plus the 174 bp *nra* upstream promoter region within wt Alab49, was substituted with a 1,494 bp *rofA* gene plus a 266 bp upstream region containing RofA-binding sites essential for autoregulation [16] (Figure 1C); the 256 bp region immediately upstream of *cpa* of wt Alab49 was preserved [10]. The *rofA*-containing chimeric construct, having a 522 bp *rofA-cpa* intergenic region, is denoted Alab49 *rofA::aad9*. The Alab49 *rofA::aad9* chimera shares the *mga2-rofA* genotype with the majority of *emm* pattern E generalist strains [5].

RNA transcript levels for the *cpa* through *fctB* series of FCT-region genes, encoding proteins necessary for pilus structure and assembly, was measured by qRT-PCR for both Alab49 *rofA::aad9* and wt Alab49 containing the *nra* gene (Table 1). The *rofA*-containing chimera displayed a substantial increase in transcript abundance relative to wt Alab49. No remarkable change in transcript levels was observed for the FCT-region genes *msmR* or *prtF2* (Table 1); ratios >2- or <0.5-fold are considered to be of probable biological significance.

**Table 1:** Effects of RofA and the modified intergenic region, on FCT-region gene transcription, as determined by qRT-PCR.

Growth phase	OD600 nm **	Transcript abundance in Alab49 *rofA::aad9*, relative to wt *						
		*cpa*	*sipA2*	*fctA*	*srtC2*	*fctB*	*msmR*	*prtF2*
mid log	0.337±0.038	**42.8**±33.4	**27.0**±20.0	**21.2**±12.7	**18.1**±3.4	**14.3**±7.1	1.49±0.69	1.38±1.25
late log	0.543±0.006	**33.9**±26.2	n.d.	**26.3**±14.3	n.d.	n.d.	n.d.	0.93±0.45
stationary	0.855±0.026	**11.5**±12.7	**19.2**±25.1	**10.2**±8.2	**7.4**±6.1	**5.7**±1.1	1.17±0.37	1.14±0.88
t-value, mid log versus stationary ˆ		0.0006	NS	0.013	0.029	0.050	NS	NS

* Each mid log and stationary phase value is based on at least 4 different RNA preparations; not all genes were tested with every RNA preparation; some measures were repeated in additional experiment(s) with new cDNA. The data for late log phase are based 3 different RNA preparations, with 1 or 2 cDNA preparations, tested for a total of 6 to 8 times. Ratios >2-fold are highlighted in bold.

** Average for each RNA preparation from Alab49 *rofA::aad9* cultures, used to generate the cumulative qRT-PCR data.

ˆ The t-value is based on the transcript abundance ratio relative to wt Alab49, for mid log versus stationary phase cultures (unpaired, 2-tailed t-test). NS, not significant; n.d., not determined.

The absence of a polar effect on transcription of the non-FCT-region gene *hsp33*, lying immediately downstream of *aad9* in the Alab49 *rofA::aad9* construct (Figure 1B), is supported by qRT-PCR findings (Table 2). The relative lack of a polar effect on *hsp33* was also reported for the similarly engineered Alab49 *nra::aad9* construct that serves as a positive control [14].

**Table 2:** Effects of RofA and the modified intergenic region, on non-FCT-region gene transcription, as determined by qRT-PCR.

Growth phase	Transcript abundance in Alab49 *rofA::aad9*, relative to wt *				
	*hsp33*	*mga*	*pam (emm)*	*speB*	*ska*
mid log	1.78±0.77	1.25±0.38	1.18±0.62	0.86±0.33	1.23±0.54
stationary	0.62±0.08	1.24±0.67	1.53±0.81	1.35±0.72	1.84±0.60

* Each value is based on at least 3 different RNA preparations.

Elevation in the level of pilus gene transcription in Alab49 *rofA::aad9* was most profound during the exponential phase of growth, ranging from ∼14- to 44-fold increases over wt (Table 1). For stationary phase cultures, the increases in pilus gene transcript abundance ranged from ∼6- to 19-fold. The differences in relative transcript abundance for mid log versus stationary phase cultures were statistically significant for most of the *cpa* through *fctB* genes. Therefore, activation of pilus gene expression in the Alab49 *rofA::aad9* construct appears to peak during exponential growth.

**Figure 2:**
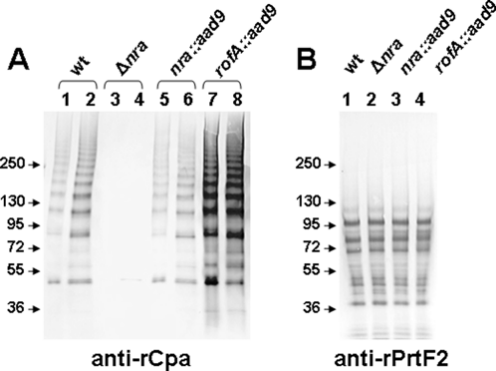
Increased quantity of pilus-like structures in the *rofA::aad9* construct. Immunoblots were treated with antiserum raised to rCpa (A) or rPrtF2 (B). Mutanolysin extracts subject to SDS-PAGE were prepared from wt Alab49 (lanes 1 and 2), Alab49 Δnra mutant (lanes 3 and 4), Alab49 *nra::aad9* (lanes 5 and 6) and Alab49 *rofA::aad9* (lanes 7 and 8). Extracts from cells grown to mid-logarithmic phase (4 h at 30°C) are shown in lanes 1, 3, 5 and 7; extracts from cells grown to stationary phase (16 h at 30°C) are shown in lanes 2, 4, 6 and 8. To account for the increase in bacterial cell mass at stationary phase, the loading volume of the samples obtained from mid log phase cultures was increased by 2.5-fold, a value which corresponds to the observed increase in OD_600_ for the stationary phase cultures relative to mid log phase. Antiserum to rFctA and rFctB show staining patterns highly similar to those observed with anti-rCpa serum (supplement; Figure S1). Because PrtF2 is completely degraded by SpeB in stationary phase cultures [17], only extracts from mid log cultures are shown (panel B); also the protease inhibitor cocktail was not included during the extraction procedure. Molecular weight markers are indicated in kilodaltons.

Consistent with the qRT-PCR findings, immunoblot analysis of cell extracts of Alab49 *rofA::aad9* showed increased immunoreactivity to proteins comprising the polymeric ladder corresponding to the pilus-associated protein Cpa (Figure 2A), but not to PrtF2 (Figure 2B). A similar increase in the quantity of pilus proteins within the Alab49 *rofA::aad9* construct was also observed using antiserum directed to recombinant pilus proteins FctA and FctB (Figure S1). The increase in pilus proteins is found among both the lower and higher molecular weight polymeric forms, hinting that increased expression may result in additional pili structures rather than longer pili, although this is a difficult measure due to pilus fragility.

As reported previously [14], the Alab49 Δnra mutant exhibits a large loss in the quantity of the pilus-associated polymeric ladder, whereas the Alab49 *nra::aad9* replacement mutant restores the wt Alab49 phenotype (Figures 2A, S1). Additionally, Alab49 Δnra is T-nontypable, whereas wt Alab49 and the Alab49 *nra::aad9* construct are T3/13/B. The T3/13/B serotype is also restored in Alab49 *rofA::aad9* (data not shown). The T-serotype is based, at least in part, on the pilus structure [11], [17].

Taken together, the data demonstrate that replacement of the kanamycin-resistance gene *aphA3* in the Δ*nra* mutant, with *rofA* plus the modified *rofA*-*cpa* intergenic region (plus *aad9*), results in the restoration of pilus gene transcription. Furthermore, the Alab49 *rofA::aad9* construct exhibits a substantial increase both in transcription of the *cpa* through *fctB* series of genes and pilus-like protein biosynthesis, relative to the Nra-dependent levels observed for the wt Alab49 strain. Importantly, RofA provides a functional substitute for Nra activation of pilus gene expression in this particular *S. pyogenes* strain background.

### Alab49 *rofA::aad9* has an elevated growth rate at the skin

The humanized mouse model for GAS impetigo provides a highly sensitive and specific measure for an infectious process that closely mimics the pathological changes observed in natural human disease [8], [17]–[21]; a drawback is that it is not high throughput. Human skin grafts present on SCID mice are gently scratched and bacteria are topically applied. Histopathology scoring for erosion of the epidermis and infiltration of neutrophils reveals a significant correlation with the net increase or decrease in colony forming units (cfus) recovered from skin grafts following tissue biopsy at 7 days (d) post-inoculation. The net change in the number of cfus, from the time of inoculation to biopsy, is the primary quantitative outcome measure for this assay and it reflects the short term fitness of the organism.

**Figure 3:**
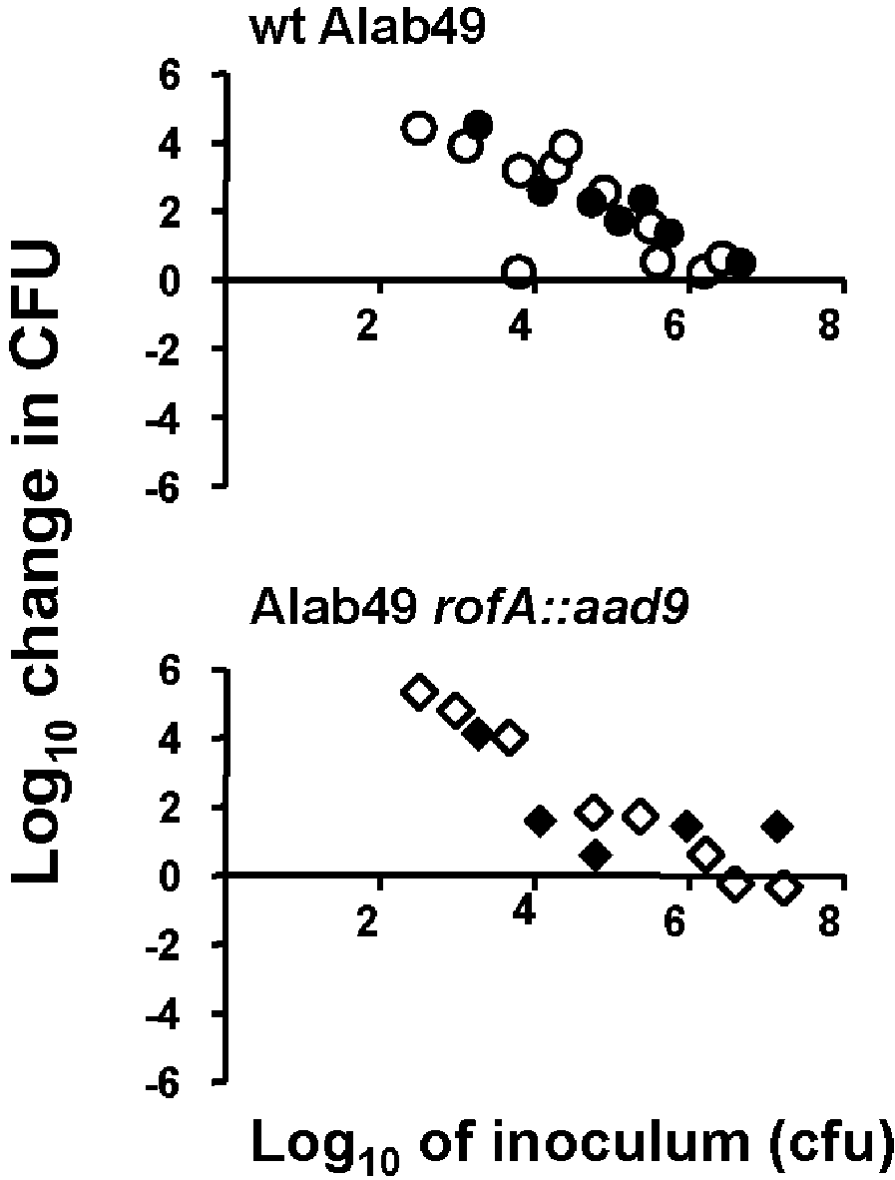
Skin infection in the humanized mouse at 7 d post-inoculation. Bacteria grown to either mid-logarithmic (filled symbols) or stationary (open symbols) phase in broth culture were used to inoculate scratched human skin engrafted on SCID mice. The inoculum dose (log_10_ CFUs) is depicted on the x-axis. The net change (increase or decrease) in log_10_ CFUs recovered from a graft at biopsy, relative to the inoculum dose, is shown on the y-axis. Each data point represents an inoculated skin graft. Bacterial inoculums are indicated as wt Alab49 (upper panel) and Alab49 *rofA::aad9* (lower panel). Differences in inoculum doses tested, for wt Alab49 versus Alab49 *rofA::aad9*, at either the exponential or stationary growth phase, are not statistically significant according to either the parametric unpaired t-test or non-parametric Mann-Whitney U test (2-tailed).

The Alab49 *rofA::aad9* construct was tested for virulence in the humanized mouse model for skin infection (Figure 3). Tissue biopsies taken at 7 d post-inoculation show that of the 13 skin grafts inoculated with Alab49 *rofA::aad9* cultures grown to either mid-log or stationary phase, 11 (85%) displayed a net increase in bacterial growth at the skin, indicative of “virulence” (Figure 3, lower panel). The differences in net growth at the skin, for wt Alab49 (Figure 3, upper panel) versus Alab49 *rofA::aad9* (lower panel), were not statistically significant. In sharp contrast, inactivation of *nra* in Alab49 led to a significant decrease in the number of cfus recovered from the skin [14]. Thus, replacement of the *nra* defect with *rofA* leads to restoration of the wt phenotype.

The total number of cfu recovered from skin grafts at 7 d for wt Alab49 versus Alab49 *rofA::aad9* was similar, irrespective of the inoculum dose, yielding an overall mean average of 2.2×10^7^ cfus (t = 0.85, for wt versus *rofA::aad9*; unpaired, 2-tailed). Since 7 d post-inoculation appears to be a saturating time point for bacterial growth at the skin by virulent strains, an earlier biopsy time was also examined.

**Figure 4:**
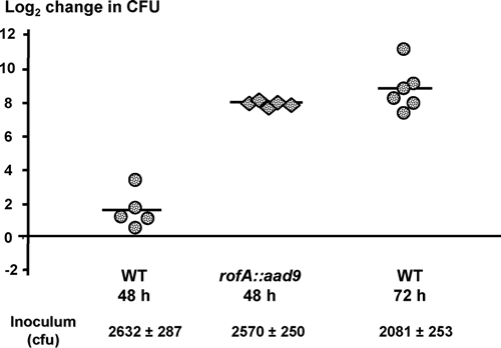
Growth rate during the early stages of skin infection. The number of bacterial population doublings (log_2_ change in cfu) at the skin by 48 or 72 h post-inoculation is shown for mid-logarithmic phase broth cultures of wt Alab49 (circles) and the Alab49 *rofA::aad9* construct (diamonds). Bars depict average mean values. The mean average inoculum dose and standard deviation is also indicated.

Equivalent doses of ∼2,500 cfus of wt and Alab49 *rofA::aad9* grown to mid-log phase were used to inoculate skin grafts that underwent biopsy 48 hours (h) later; with this size inoculum, approximately 13 population doublings are projected for saturated bacterial growth at the skin. At 48 h, wt Alab49 had undergone a mean average of 1.7±1.1 population doublings (Figure 4). In contrast, Alab49 *rofA::aad9* had undergone 8.0±0.2 population doublings by 48 h. The difference between wt Alab49 and *rofA::aad9* for population doubling at the skin at 48 h is highly significant (t<0.01; unpaired t-test, 2-tailed). Thus, the *rofA::aad9* construct displays greater short term fitness at the skin than the wt.

As a control, the bacterial growth curves in enriched broth were found to be nearly identical for wt Alab49 and the *rofA::aad9* construct (data not shown).

In summary, the Alab49 *rofA::aad9* chimera, which over-expresses the polymeric pilus proteins, displays a higher growth rate during the initial stages of infection at the skin.

### Pilus polymer quantity does not strictly correlate with growth rate at the skin

The higher growth rate of Alab49 *rofA::aad9* at the skin relative to wt (Figure 4), and the loss in cfus with the Alab49 Δ*nra* mutant [14], is consistent with a direct correlation between the amount of polymeric pilus protein produced (Figure 2A) and bacterial growth at the skin. However, a previous study showed that the Alab49 Δ*fctA* mutant undergoes a sharp reduction in the amount of pilus-associated proteins present in a polymeric form, yet the Δ*fctA* mutant behaves similar to wt Alab49 in terms of net growth at the skin at 7 d post-inoculation [17].

To more precisely address whether the quantity of pilus polymer corresponds to the differences in bacterial growth at the skin, Δ*fctA*-infected grafts were examined at earlier time points, before saturated growth was attained; inoculums were prepared from mid log phase cultures. At 48 h post-inoculation, the Δ*fctA* mutant underwent an average of 1.1±2.0 population doublings (Figure S2). The difference between the Δ*fctA* mutant and wt Alab49 (Figure 4) was not statistically significant (unpaired, t-test; 2-tailed). This finding confirms, in a more rigorous manner, that the amount of polymeric pilus protein does not strictly correlate with bacterial growth at the skin. At 72 h post-inoculation, the average number of population doublings was 8.9±1.3 and 10.0±0.1 for wt Alab49 and the Δ*fctA* mutant, respectively, which more closely approximates the values for the faster replicating Alab49 *rofA::aad9* mutant after only 48 h of skin infection (Figures 4 and S2).

The findings on the Alab49 Δ*fctA* mutant support the notion that the effect of RofA on bacterial growth at the skin is not dependent on the pilus structure. Key factors underlying the altered biological phenotype may be virulence factors encoded by genes outside of the FCT-region. Alternatively, FCT-region proteins that are not necessarily in a pilus form may be critical, such as Cpa, whose expression is enhanced in Alab49 *rofA::aad9* (Table 1) and which plays a crucial role in virulence in the humanized mouse when inoculated during logarithmic phase, despite loss of the polymeric pilus structure in the Δ*cpa* mutant [17].

### Effect of Alab49 *rofA::aad9* on non-FCT region genes

The effect of replacement of *nra* with *rofA* on transcription outside the FCT region was assessed for genes previously shown to be essential for Alab49 virulence in the humanized mouse [18], [19], [21]. No difference in the abundance of *mga*, *pam*, *speB* or *ska* transcripts in Alab49 *rofA::aad9* was observed, relative to wt Alab49 (Table 2). As further confirmation, tests for secreted cysteine protease and bacterial-bound plasmin activity showed no significant difference between *rofA::aad9* and wt (data not shown). The quantity of hyaluronic acid capsule, a key virulence factor whose regulation of expression is complex, was also close to equivalent for the *rofA* chimeric construct, yielding 18.0 and 20.2 fg of hyaluronic acid per cfu for wt Alab49 and *rofA::aad9*, respectively. The data indicate that in strain Alab49, the substitution of *nra* with *rofA* has no detectable effect on several other key virulence phenotypes that are known to be critical for skin infection by GAS.

### Transcriptional start sites in the Alab49 *rofA::aad9* construct

The possible mechanisms by which pilus gene transcription is enhanced in the Alab49 *rofA::aad9* chimeric construct were examined in greater depth. The 522 bp *rofA-cpa* intergenic region of Alab49 *rofA::aad9* has an additional set of potential promoter sites for pilus gene transcription (Figure 1C), located within the 266 bp region derived from strain D471 (M6); this promoter is utilized in the transcription of the *prtF1* gene, which shares a locus comparable in position to that of the *cpa* gene in Alab49. In order to determine whether the P*prtF1* site of the *rofA-cpa* intergenic region contributes to transcription of pilus genes, oligonucleotides targeting the *cpa* 5′-untranslated region were used to better localize the transcriptional start site.

**Figure 5:**
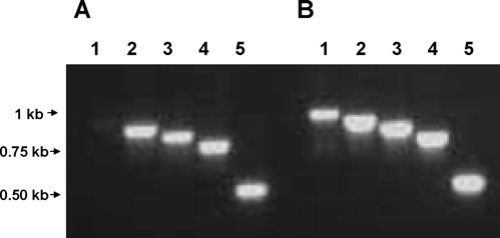
Start site for pilus gene transcription in the *rofA::aad9* construct. cDNA generated from RNA derived from wt Alab49 (A) or Alab49 *rofA::aad9* (B) was used as a template for PCR amplification; amplicons were subject to agarose gel electrophoresis and ethidium bromide staining. All reactions are paired with reverse primer CPA-R in combination with UP-F1 (lane 1), UP-F2 (lane 2), UP-F3 (lane 3), UP-F4 (lane 4) and CPA-F (lane 5). Positions for primers UP-F1 through F4 are also depicted in Figure 1C. Molecular size markers are indicated.

The UP-1 PCR amplification includes a forward primer that matches the start of the 256 bp *cpa* upstream region, lying at a position equivalent to the *prtF1* start codon in the M6 strain from which the 266 bp region immediately upstream of *rofA* was derived (Figure 1C); the paired reverse primer hybridizes within the *cpa* ORF. Using cDNA generated from purified RNA as the template for PCR, wt Alab49 failed to yield an amplification product with the UP-F1 primer (Figure 5A, lane 1), whereas the Alab49 *rofA::aad9* construct yielded an amplicon of the expected size (Figure 5B, lane 1). Furthermore, genomic DNA from both wt and *rofA::aad9* yielded a PCR product with this primer pair (data not shown), showing that the genomic DNA sequences are similar in this region. The results indicate that the P*prtF1* site may contribute to the elevated level of pilus gene transcription in the Alab49 *rofA::aad9* construct (Table 1).

To further localize the *cpa* transcriptional start site in wt Alab49, additional oligonucleotide primers were tested for PCR amplification of the *cpa* 5′-untranslated region. The UP-2 priming site lies downstream from a putative MsmR-binding site and P*cpa* site [12], [22] (Figure 1C). In contrast to UP-1, primer UP-2 yielded amplicons using cDNA templates derived from both wt Alab49 and the *rofA::aad9* construct (Figure 5; lanes 2), indicating that the *cpa* transcriptional start site within wt Alab49 lies upstream of the UP-2 priming site.

That RofA mediates enhanced transcription of the *cpa* 5′-untranslated region is supported by qRT-PCR results, comparing wt to *rofA::aad9*, with both the UP-3 and UP-4 priming reactions (Table 3; Figure 1C).

**Table 3:** Effects of RofA and the modified intergenic region, on transcript levels for the *cpa* 5′-untranslated region in Alab49, as determined by qRT-PCR.

Growth phase	Transcript abundance of Alab49 *rofA::aad9*, relative to wt Alab49	
	UP-3	UP-4
mid log	**151.4**±46.3	**55.7**±21.5
late log	**48.9**±34.8	**23.9**±18.0

* Each value is based on 2 or 3 different RNA preparations generated in parallel, with 1 or 2 cDNA preparations generated from each RNA preparation, for a total of 3 or 4 experiments, each performed with duplicate or triplicate wells. Ratios >2-fold are highlighted in bold.

Precise localization of the *cpa* transcriptional start sites in wt Alab49 and the *rofA::aad9* construct was achieved by rapid amplification of cDNA ends (RACE). Using a 5′end phosphorylated oligonucleotide primer hybridizing within the *cpa* ORF, the *cpa* transcriptional start site was mapped to 214 bp upstream from the TTG start codon in wt Alab49 (Figure 1C). In contrast, the *cpa* transcriptional start site was 275 bp upstream from the start codon in the Alab49 *rofA::aad9* construct. The data show that RofA does not activate *cpa* through its native promoter, but rather, requires the P*prtF1* site of the original M6 strain from which it was derived.

### Regulatory effects of MsmR within the *rofA* chimeric construct

The 256-bp region upstream of the *cpa* ORF contains two putative MsmR-binding sites (Figure 1C) that differentially overlap with the transcriptional start sites in wt Alab49 and the *rofA::aad9* construct (Figure 5). MsmR has a slight repressive effect on *nra* and pilus gene transcription in wt Alab49 [14]. To gain further insight on the mechanism underlying altered pilus gene expression in the Alab49 *rofA::aad9* construct, the *msmR* gene was inactivated to generate Alab49 *rofA::aad9* Δ*msmR*.

In contrast to *nra* in wt Alab49 [14], there is no measurable change in *rofA* transcription when the *rofA::aad9* Δ*msmR* mutant is compared to isogenic Alab49 *rofA::aad9* (Table 4). Thus, *rofA* transcription is unaffected by MsmR in the Alab49 *rofA::aad9* chimera. But instead of MsmR-mediated repression of pilus gene transcription, the relative abundance of *cpa* transcript is reduced by slightly more than 2-fold in the *rofA::aad9* ΔmsmR mutant for cultures grown to mid log phase. The data suggest that MsmR may have a slight activating effect on *cpa* transcription in the *rofA*-harboring construct, and this effect appears to be independent of changes in *rofA* expression levels.

**Table 4:** Effects of MsmR on gene transcription in the chimeric construct Alab49 *rofA::aad9* Δ*msmR*, as determined by qRT-PCR.

Growth phase	OD600 nm **	Transcript abundance in Alab49 *rofA::aad9* Δ*msmR*, relative to Alab49 *rofA::aad9* *				
		*rofA*	*cpa*	*fctA*	*prtF2*	*mga*
mid log	0.316±0.0.29	0.91±0.37	**0.48**±0.22	0.60±0.18	**0.051**±0.017	0.73±0.14
late log	0.554±0.009	0.90±0.33	0.63±0.22	0.82±0.28	**0.004**±0.006	n.d.
stationary	0.867±0.013	1.67±0.24	1.48±0.71	1.38±0.40	**0.138**±0.081	0.84±0.39

* Each mid log and stationary phase value is based on 3 different RNA preparations generated in parallel, with 2 cDNA preparations generated from each RNA preparation (6 experiments in total), except for *mga* (based on 4 experiments). The data for late log phase are based 3 different RNA preparations, with 1 or 2 cDNA preparations, tested for a total of 5 to 8 times. Ratios <0.5-fold are highlighted in bold.

** Average for each RNA preparation from Alab49 *rofA::aad9* Δ*msmR* cultures, used to generate the cumulative qRT-PCR data.

**Figure 6:**
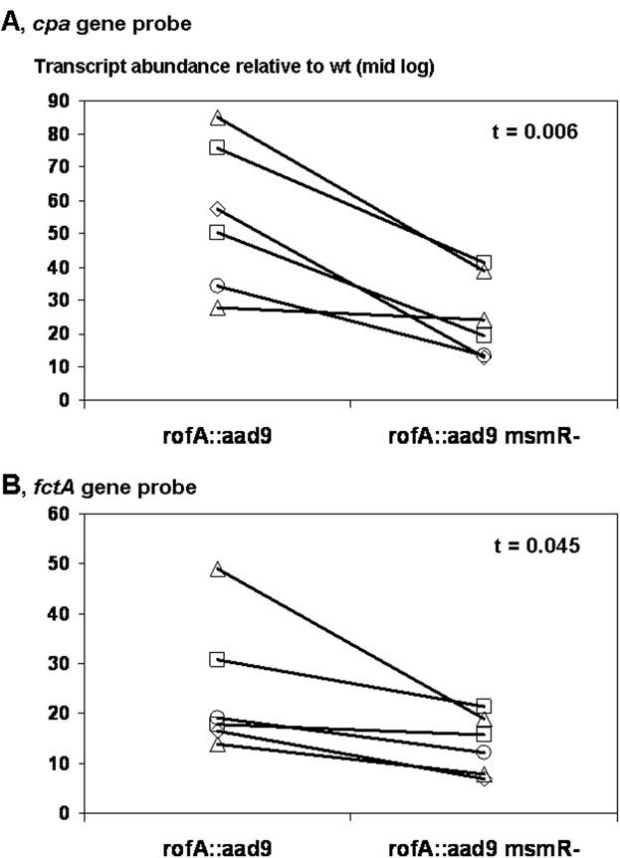
Decrease in pilus gene transcript levels in *rofA::aad9* Δ*msmR*. qRT-PCR data showing transcript abundance for *rofA::aad9* and *rofA::aad9* Δ*msmR* relative to wt Alab49, for *cpa* (panel A) and *fctA* (panel B). RNA was prepared from three bacterial cultures - wt, *rofA::aad9* and *rofA::aad9* Δ*msmR* - grown to the mid-logarithmic phase in three separate experiments; two independent cDNA preparations were generated from each RNA preparation. Matched pairs of strains from the six different cDNA preparations are plotted. Statistical significance is measured for transcript ratios of *rofA::aad9* versus *rofA::aad9* Δ*msmR* by the paired t-test (2-tailed).

The activation of pilus gene transcription by MsmR becomes even more apparent when the relative transcript abundance of each *rofA*-containing construct is calculated based on comparison to wt Alab49 (Figure 6). In each of six experiments using bacteria grown to mid log phase, a reduction in the relative ratio of *cpa* and *fctA* transcript abundance is observed for the *rofA::aad9* Δ*msmR* mutant as compared to Alab49 *rofA::aad9*. Using the paired t-test, the ratio of pilus gene transcript levels is significantly lower for the *rofA::aad9*Δ*msmR* mutant as compared to the Alab49 *rofA::aad9* construct (t = 0.006 for *cpa* and t = 0.045 for *fctA*; 2-tailed).

**Figure 7:**
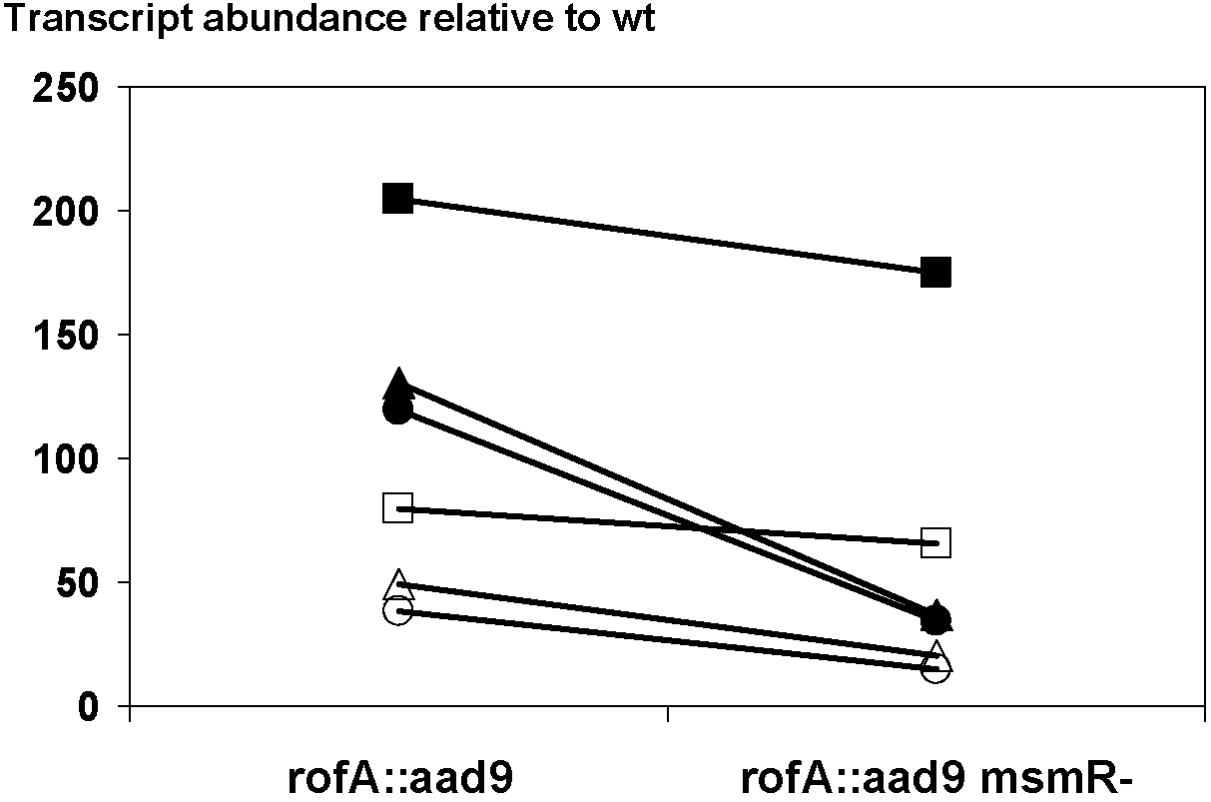
MsmR effects on transcripts corresponding to the 5- untranslated region of *cpa*. qRT-PCR data showing transcript abundance for *rofA::aad9* and *rofA::aad9* Δ*msmR* relative to wt Alab49, for reactions using UP-F3 (filled) and UP-F4 (open) primers targeting the 5′-untranslated region of *cpa* (Figure 1C). RNA was prepared from bacterial cultures grown to the mid-logarithmic phase in three separate experiments.

The reversal in polarity of MsmR effects on pilus gene expression in Alab49 harboring *nra* (wt) versus *rofA* (*rofA::aad9*) is also evident when the 5′-untranslated region upstream of *cpa* is evaluated by qRT-PCR. Both the UP-3 and UP-4 primer sets show an increase in transcript abundance of ∼2- to 4-fold when the Δ*msmR* mutant is compared to wt Alab49 (Table 5), adding further support to the previous finding that MsmR acts as a repressor of *cpa* transcription in Alab49 containing *nra* [14]. In contrast, the UP-3 region is diminished in abundance by slightly >2-fold when the *rofA::aad9* Δ*msmR* mutant is compared to the *rofA::aad9* construct. Furthermore, in each of three experiments, the ratio of UP-3 and UP-4 transcripts, relative to wt Alab49, is consistently lower for the *rofA::aad9* Δ*msmR* mutant versus Alab49 *rofA::aad9* (Figure 7).

**Table 5:** Effects of MsmR on transcript levels for the *cpa* 5′-untranslated region in Alab49, as determined by qRT-PCR.

	Transcript abundance in Alab49 Δ*msmR*, relative to wt Alab49 *		Transcript abundance in Alab49 *rofA::aad9* Δ*msmR*, relative to Alab49 *rofA::aad9*	
Growth phase	UP-3	UP-4	UP-3	UP-4
mid log	**4.22**±1.93	**2.24**±0.62	**0.47**±0.33	0.54±0.25

* Each value is based on 3 different RNA preparations. Ratios <0.5-fold or >2-fold are highlighted in bold.

**Figure 8:**
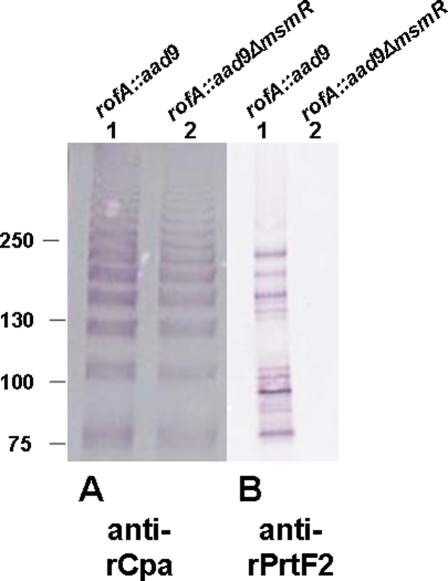
Effect of MsmR on extracted pilus and PrtF2 proteins. Immunoblots were treated with antiserum raised to rCpa (A) or rPrtF2 (B). Mutanolysin extracts subject to SDS-PAGE were prepared from Alab49 *rofA::aad9* (lanes 1) or Alab49 *rofA::aad9* Δ*msmR* (lanes 2). Extracts from cells grown to stationary phase (16 h at 30°C) are shown in panel A; extracts from cells grown to mid-logarithmic phase (4 h at 30°C) are shown in panel B. Molecular weight markers are indicated in kilodaltons.

The slight activating effect of MsmR in the *rofA*-positive background is further substantiated by immunoblot analysis (Figure 8A), where a slight decrease in the polymeric ladder-like structure is observed for the *rofA::aad9* Δ*msmR* mutant.

Unlike pilus gene transcription, the effect of MsmR in wt Alab49 harboring *nra* [14] versus the *rofA::aad9* Δ*msmR* mutant is highly similar for *prtF2* transcription. The qRT-PCR data obtained with the *rofA::aad9* Δ*msmR* mutant demonstrates that MsmR is a potent activator of *prtF2* transcription (Table 4). For mid log phase cultures, *prtF2* transcript levels are depressed ∼20-fold in *rofA::aad9* Δ*msmR*, relative to the Alab49 isogenic *rofA::aad9* construct. Alterations in *prtF2* transcript levels are further substantiated by immunoblot analysis, wherein the *rofA::aad9* Δ*msmR* mutant lacks material that is immunoreactive with antiserum raised to recombinant PrtF2 (Figure 8B).

In summary, data show that MsmR has a slight activating effect on pilus gene expression in Alab49 *rofA::aad9*. This is in contrast to its repressor function in wt Alab49 harboring *nra* [14]. Despite opposite effects of MsmR on the polarity of pilus gene expression in *nra*-positive versus *rofA*-positive Alab49 strains, MsmR is a strong activator of *prtF2* gene expression in both genetic backgrounds.

### Pilus gene regulation in Alab49 *rofA::aad9* lacking MsmR

Since MsmR functions as an activator of pilus gene expression in the *rofA* chimeric construct, it was of interest to examine transcription in the absence of *msmR*. This was achieved by comparing relative pilus gene transcript abundance of Alab49 *rofA::aad9* Δ*msmR* to Alab49 Δ*msmR* (Table 6). In the absence of MsmR input function, both *cpa* and *fctA* transcript levels were markedly elevated in the RofA-harboring construct, relative to the Nra-positive strain. Thus, the data indicate that enhancement of pilus gene transcription in Alab49 *rofA::aad9* is not wholly dependent on MsmR; this finding is not unexpected since the activating effect of MsmR is only slight (Tables 4 and 5).

**Table 6:** Effects of RofA and the modified intergenic region, on FCT-region gene transcription in the absence of *msmR*, as determined by qRT-PCR.

Growth phase	OD600 nm **	Transcript abundance in Alab49 *rofA::aad9* Δ*msmR*, relative to Alab49 Δ*msmR* *	
		*cpa*	*fctA*
mid log	0.316±0.0.29	**11.93**±8.86	**2.42**±1.18
stationary	0.867±0.013	**8.35**±2.92	**5.30**±1.16
t-value, mid log versus stationary ˆ		NS	0.030

* Each mid log and stationary phase value is based on 3 different RNA preparations generated in parallel, with 2 cDNA preparations generated from each RNA preparation (6 experiments in total). Differences >2-fold are highlighted in bold.

** Average for each RNA preparation from Alab49 *rofA::aad9* Δ*msmR* cultures, used to generate the cumulative qRT-PCR data.

ˆ t-value is based on the transcript abundance ratio relative to wt Alab49, for mid-logarithmic versus stationary phase cultures (paired, 2-tailed t-test). NS, not significant.

Strikingly, the logarithmic growth phase peak for enhanced pilus gene expression in the Alab49 *rofA::aad9* chimera (Table 1) appears to be abolished in the absence of MsmR (Table 6). Non-significant differences between mid log versus stationary phase cultures were observed for the elevated levels of *cpa* transcript, relative to Alab49 Δ*msmR*, whereas the elevated levels of *fctA* transcript were significantly higher at stationary phase.

Taken together, the data support the idea that the MsmR-mediated component of pilus gene transcriptional activation in Alab49 harboring *rofA* exerts its effect during exponential growth. Furthermore, the logarithmic growth phase peak for pilus gene transcription in Alab49 *rofA::aad9* is dependent on MsmR. In contrast, the MsmR-mediated repression of *nra* and pilus gene transcription in the wt Alab49 background has no apparent growth phase dependence [14].

## Discussion

Member organisms of the *S. pyogenes* species exhibit diversity in their preferred ecological niche for reproductive growth and transmission, which for many strains is either the infected throat or skin of the human host. The strong genetic linkage disequilibrium observed between the *nra-* versus *rofA-*lineage transcriptional regulatory genes, and genotypes for preferred tissue site of infection [5], is highly suggestive of a direct link between different gene expression programs and tissue-specific environmental signals and/or adaptive strategies.

The divergent RofA plus upstream region is a functional substitute for Nra in terms of activation of pilus gene expression, although the magnitude of the activating effect is markedly enhanced. Surprisingly, the RofA-containing construct of Alab49 exhibited higher short term fitness at the skin, relative to wt Alab49 harboring Nra. The molecular mechanism underlying the RofA-mediated effect may be ascribed to higher levels of Cpa production, a putative collagen binding protein that can be incorporated into the pilus structure [11], [17], [23]. Conceivably, undefined genes lying outside the FCT-region might also be regulated by RofA [24] in Alab49 and thereby, influence GAS infectivity at the skin. However, the amount of pilus polymer produced, as measured by immunoblot, is not a critical determinant of bacterial growth in the humanized mouse. Since bacteria are applied directly to wounded skin, a functional role for pili in transmission and/or the very early stages of a newly acquired infection may be bypassed.

The finding of increased short term fitness at the skin by the Alab49 chimera harboring RofA, the orthologous form usually associated with throat specialist and generalist strains of *S. pyogenes*, was not anticipated. One possible explanation is that the “hypervirulence” detected in the humanized mouse model for impetigo is not evolutionarily favored over the long term in a natural host population. Notably, a higher rate of bacterial growth in the tissue may reduce the length of the time period for infectiousness, leading to a reduction in the basic reproductive rate, Ro [25], [26]. During the natural course of GAS skin infection in humans, the purulent impetiginous lesion transitions to scab formation and a dried out, healed state that is unfavorable for bacterial survival. It would also be interesting to establish whether the Alab49 *rofA::aad9* chimera has enhanced virulence at the throat, but this must await development of an animal model for pharyngitis of comparable sensitivity and specificity.

The transcriptional regulatory network of *S. pyogenes* exhibits many differences among strains [5], [15], [27]. The presence versus absence of transcription regulatory (TR) genes and target genes (TG) dictate which connections are even possible. Sequence polymorphisms within those genes/products and *cis* elements can also have a profound effect on the quality and strength of the TR-TG connections. Furthermore, the nature and strength of the TR-TG connections are dependent on spatial-temporal factors, such as the relative concentrations of each of the interacting regulatory proteins over time.

**Figure 9:**
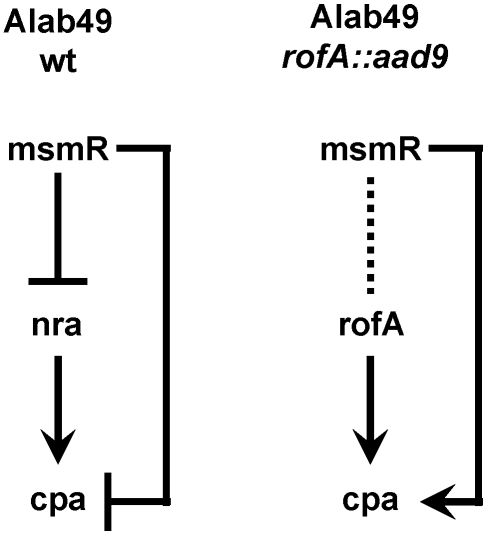
Network motifs involving *msmR*, *nra/rofA* and *cpa*. Pathways of the circuitry are shown for wt Alab49 [14] and the Alab49 *rofA::aad9* construct (this report). Positive and negative regulation is represented by arrows and bars, respectively.

The substitution of *nra* with *rofA*, and/or the 430 bp nt *nra-*cpa intergenic region with the 522 bp *rofA-*cpa intergenic region, results in a marked change in the strength of the TR-TG connection between the Nra/RofA regulator and the pilus gene target, evidenced by a dramatic increase in the relative ratio of pilus gene transcript abundance. The growth phase peak for this enhanced transcriptional activity (i.e., log phase) in the RofA-positive construct is dependent on MsmR, indicating that MsmR modulates the synchronization of the TR-TG interaction. The regulatory effect of MsmR on the pilus gene target is opposite in the Nra versus RofA background (i.e., repression versus activation), indicative of an important shift in the qualitative nature of the TR-TG connection. The *msmR*, *nra* and *cpa* genes form a feedforward loop network motif in wt Alab49 (Figure 9) [14], [28]. In contrast, a connection between *msmR* and *rofA* is not detected in the Alab49 *rofA::aad9* chimera, and the *msmR-cpa* connection is reversed in polarity relative to the wt.

Many previous studies on Nra function and *nra* gene regulation were performed with M-type 49 (M49) organisms [12], [15], [22], [27], [29], in which Nra acts as a repressor of pilus gene transcription. Despite this striking reversal in polarity, the predicted *nra* protein products of Alab49 and the M49 strain differ at only a single amino acid residue, via an Asn-Lys conserved substitution. Yet, both Nra and RofA present in the Alab49 strain background function as activators of pilus gene expression. Thus, not only is the connection between the TR gene locus (*nra/rofA*) and the pilus TG maintained following orthologous gene replacement in Alab49, but the qualitative nature of that connection (i.e., activation) is also preserved, despite the high level of sequence divergence (∼35%) between Nra and RofA. Experimental manipulation of the TR genes, as demonstrated in this report, underscores the plasticity and robustness of the circuitry in complex regulatory networks.

Not only were the ORFs of *nra* and *rofA* swapped in the Alab49 background, but so were their divergent upstream regions, which are required for auto-regulatory control [12], [16]. Thus, the differences ascribed to wt Alab49 versus the *rofA::aad9* construct may be due directly to RofA activity, *cis* elements and/or other regulatory factors having input function. In addition to MsmR and Nra/RofA, the transcription regulatory proteins Mga and RALP3 affect transcription of FCT-region genes in at least some strains [27], [29], [30]. However, Alab49 lacks the *ralp3* gene and there is no evidence for cross-regulatory effects between Nra and Mga [14], [21], as observed for the M49 strain. Genes encoding Mga, Nra/RofA and the RALPs share a common distant ancestor, and probably evolved by gene duplication and divergence; during evolution, auto-regulation may have given rise to cross-regulation. Other yet-to-be characterized regulators might also influence gene transcription in the FCT-region of Alab49.

The importance of orthologous gene replacements in bacterial evolution remains to be fully established. In studies analogous to the work presented on *S. pyogenes*, the experimental replacement of the *Escherichia coli* regulatory gene *pmrD*, with a divergent form derived from *Salmonella enterica*, results in differential regulation of homologous genes and alterations in a probable niche-determining phenotype [31].

Unlike many bacterial pathogens, *S. pyogenes* are devoid of pathogenicity islands [32], perhaps because the high recombination rate renders genomic islands unstable. An important evolutionary advantage of recombination is that it allows for a quick exploration of a wide array of genotypes. The vast majority of mutations in bacteria are deleterious, or quickly lost from the population due to random genetic drift. Yet, the Alab49 *rofA::aad9* construct acquired a higher level of (short term) fitness at the skin as a result of orthologous gene replacement. Thus, an intermediate genotype harboring *rofA* in place of *nra* may be more likely to undergo positive selection and persist, and thereby become more readily available as the recipient of a subsequent genetic event. In general terms, orthologous genes evolve in separate species over long periods and adapt to different ecological niches. Therefore, orthologous gene replacements may play a critical role in the evolution of *S. pyogenes*, ultimately leading to quantum leaps in phenotype.

## Materials and Methods

### Bacterial culture

Unless otherwise specified, the M53 strain Alab49 and its isogenic mutants were grown at 30 or 37°C with 5% CO_2_ in Todd-Hewitt broth supplemented with 1% yeast extract (THY). Bacterial growth was monitored at OD_600_ nm.

### Mutant construction

Mutants were constructed by allelic exchange mutagenesis following transformation of bacteria with purified linear DNA containing the kanamycin or spectinomycin resistance genes (*aphA3* and *aad9*, respectively). Linear DNA cassettes were constructed by PCR-based fusion assembly and used to transform Alab49 by electroporation [17]. Primers used for construction of mutants are listed in Table S1. Transformants were selected on THY-blood agar plates containing 500 µg/ml of kanamycin or 200 µg/ml of spectinomycin, and evaluated for replacement of the target gene by PCR-based mapping and nt sequence determination. All mutants of Alab49 were confirmed to have growth curves in THY broth identical to that of wt Alab49.

Chromosomal replacement in the Δ*nra* mutant, generating the *rofA::aad9* construct, was achieved following exchange of the *aphA3* gene of the Δ*nra* mutant with the intact *rofA* gene along with its upstream region, derived from M6 strain D471, plus a spectinomycin resistance gene (*aad9*) [33] positioned downstream, as depicted in Figure 1. Construction of the Alab49 Δ*nra*, Δ*msmR* and Δ*fctA* mutants and Alab49 *nra::aad9* were previously described [14], [17]. Generation of the Alab49 *rofA::aad9* Δ*msmR* construct was achieved by replacement of *msmR* with *aphA3* on the *rofA::aad9* background, using methods previously described [14].

### Purification of RNA

Bacteria were grown overnight in THY broth, then diluted 1∶100 in fresh THY broth and grown to mid-logarithmic, late log or stationary phase. RNA was purified as previously detailed [14]. Samples were treated with DNAse I using the RNAse-free DNAse set (Qiagen) to remove potential traces of DNA in the sample; the absence of contaminating DNA was verified by failure to amplify the purified RNA samples prior to cDNA synthesis, using Taq polymerase and oligonucleotide primers targeting *recA*. The A_260_/A_280_ ratio of each RNA sample was measured to determine concentration and assess purity (i.e., ratio >1.8).

### Quantitative real time-PCR (qRT-PCR)

cDNA was synthesized from 1 µg RNA using the SuperScript III First-Strand Synthesis Supermix for qRT-PCR with random oligonucleotide primers (Invitrogen), as described previously [14]. Primers for qRT-PCR (Table S2) were designed to amplify internal regions (72 to 139 bp) within the ORFs of selected genes or 5′-untranslated regions. The *recA* gene was used as an internal reference transcription control to normalize expression data for each target gene. Each gene target was tested in duplicate or triplicate, as specified, from ≥2 RNA templates prepared from independent bacterial cultures. Relative expression of each gene was determined by the 2^−(ΔΔCT)^ method [34]. A difference >2-fold or <0.5-fold in transcript abundance was chosen as the threshold value for alterations that are likely to be biologically significant.

### Non-quantitative PCR amplification

Genomic DNA and cDNA, generated from purified RNA as described above, were used as templates for PCR amplification with Taq polymerase according to standards methods [10]. Oligonucleotide primers corresponding to the region upstream of the predicted cpa ORF were paired with CPA-R (5′-CCC CGT TGC AAT ATC AGG TTC TAT ATT ATC ACC ATA ATC ATA ACT ATC CGG CGG); they include UP-F1 (5′-GAA TTC ACT ATT AGA ATA GC), UP-F2 (5′-GAA GGA CCC CAA ATG ACA TAA TC), UP-F3 and UP-F4 (Table S2). Primer CPA-R was also tested in combination with CPA-F (5′- GAA GGT GAC TAC TCT AAA CTT CTA GAG GGA GCA AC), to amplify an internal fragment of the *cpa* gene.

### Rapid amplification of cDNA ends (RACE)

5′-end RACE was performed using a kit (TaKaRa Bio Inc.) according to the manufacturer's instructions. Oligonucleotide primers used are: RACERT, 5′-(P)CGA ACG CTC TGA TAG; RACES1, 5′-GGA AGC GCT AAC AAC AAA CG; RACES2, 5′-CGA CGA TCG GAT TAC TGA AAG; RACEA1, 5′-GCA ATA TAT CCT CTC CCT CC; and RACEA2, 5′-CCA CAT AAC AGA CTT AGC TGT C. Primer RACERT hybridizes to sequences corresponding to the *cpa* ORF and was used with reverse transcriptase for cDNA synthesis from a purified RNA template. Primers RACEA1 and RACES1 were used for the first round of PCR amplification, and RACEA2 and RACES2 were used for the second round. The final PCR-generated amplicons were purified and underwent nt sequence determination.

### Mutanolysin extraction

Cell wall extracts of GAS were prepared using mutanolysin as described [17]; unless otherwise specified, cells were harvested following growth at 30°C to mid-logarithmic phase (OD_600_ = 0.350±0.05) or stationary phase (16 h).

### Immunoblots

SDS-PAGE was performed on gradient gels (4 to 15% acrylamide) under reducing conditions. Rabbit sera raised to recombinant fusion polypeptides [17] prepared in *E. coli* and originally derived from strain Alab49 was used at a dilution of 1∶1000.

### T agglutination test

The T agglutination test was performed following trypsin treatment of whole bacteria, as described previously [17].

### Humanized mouse model for impetigo

The human skin-SCID mouse model for streptococcal impetigo was implemented as previously described in extensive detail [14], [17]–[21]. Briefly, human neonatal foreskin was engrafted onto the hind flanks of C.B.-17 *scid* mice, which fail to reject the xenografts. Healed skin grafts were gently scratched with a scalpel blade and inoculated with 50 µl of bacteria in THY broth. The inoculated bacteria had been freshly grown to mid-logarithmic or stationary phase and diluted as appropriate. The actual inoculum doses were ascertained by serial dilutions performed in duplicate and the number of cfus averaged. Mid-logarithmic phase was defined as the point of ∼half-maximal OD_600_. Stationary phase cultures were incubated for 24 h. Inoculated skin grafts were occluded with a bandage. At specified time points post-inoculation, the human skin grafts were surgically removed from mice, split and each portion weighed. One weighed portion of the graft was evaluated for the number of cfus released following a vigorous vortex, with serial dilutions performed in duplicate.

### Hyaluronic acid (HA) content

The HA content of GAS, attributable to the polysaccharide capsule, was measured according to previously described methods [18].

### Statistical analysis

Statistical significance was calculated using the t-test, either paired or unpaired, as specified, or the Mann-Whitney U-test (all tests are two-tailed). Both tests are conservative and may slightly underestimate the significance of differences between groups having a small sample size.

## Supporting Information

Figure S1 Immunoblots of bacterial cell extracts. Mutanolysin extracts were prepared from wt Alab49 (lanes 1 and 2), Alab49 Δ*nra* mutant (lanes 3 and 4), Alab49 *nra::aad9* construct (lanes 5 and 6), and the Alab49 *rofA::aad9* construct (lanes 7 and 8). Immunoblots following SDS-PAGE were reacted with antiserum raised to rFctA (panel A) or rFctB (panel B). Extracts from cells grown to mid-logarithmic phase (4 h at 30°C) are shown in lanes 1, 3, 5 and 7; extracts from cells grown to stationary phase (16 h at 30°C) are shown in lanes 2, 4, 6 and 8. Molecular weight markers are shown (kDal).
(0.20 MB TIF)

Figure S2 Absence of assembled pili has no effect growth rate at the skin during early stages of infection. The number of bacterial population doublings (log2 change in cfu) at the skin by 48 or 72 h post-inoculation with mid-logarithmic phase broth cultures of the Alab49 ΔfctA mutant (diamonds). Bars depict average mean values. The mean average inoculum dose and standard deviations are also indicated.
(0.06 MB TIF)

Table S1 (0.08 MB PDF)

Table S2 (0.08 MB PDF)
