# Supplementary figures and images for: Impact of Orthologous Gene Replacement on the Circuitry Governing Pilus Gene Transcription in Streptococci
Source: PLoS One. 2008 Oct 20;3(10):e3450. doi: 10.1371/journal.pone.0003450 (PMC2565503; doi:10.1371/journal.pone.0003450)

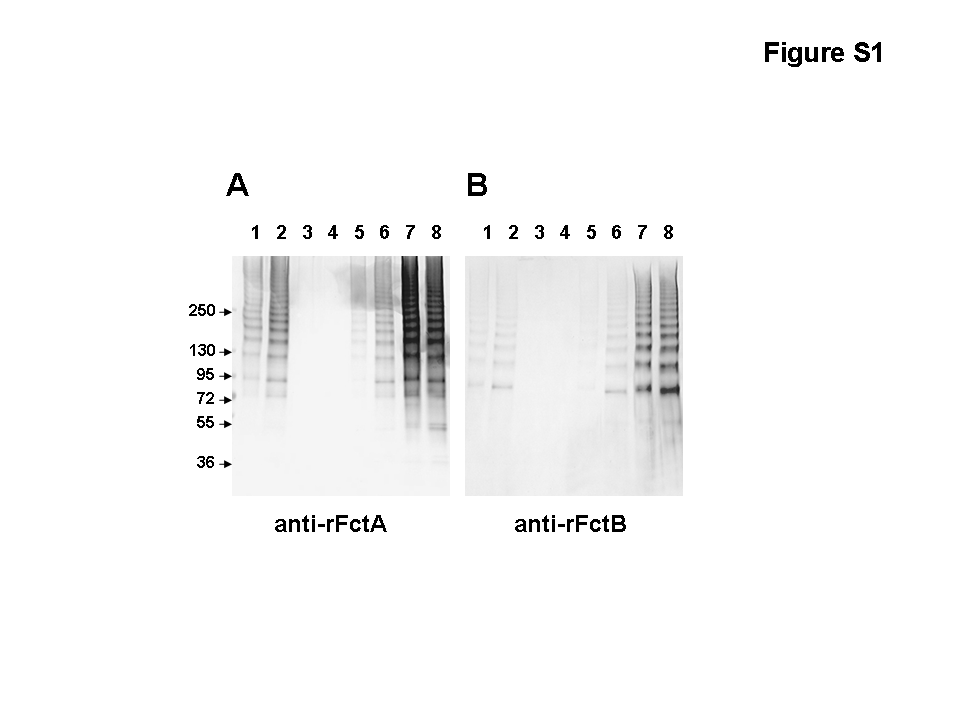

Supplement: Figure S1 — Immunoblots of bacterial cell extracts. Mutanolysin extracts were prepared from wt Alab49 (lanes 1 and 2), Alab49 Δnra mutant (lanes 3 and 4), Alab49 nra::aad9 construct (lanes 5 and 6), and the Alab49 rofA::aad9 construct (lanes 7 and 8). Immunoblots following SDS-PAGE were reacted with antiserum raised to rFctA (panel A) or rFctB (panel B). Extracts from cells grown to mid-logarithmic phase (4 h at 30°C) are shown in lanes 1, 3, 5 and 7; extracts from cells grown to stationary phase (16 h at 30°C) are shown in lanes 2, 4, 6 and 8. Molecular weight markers are shown (kDal). (0.20 MB TIF) [file pone.0003450.s001.tif]

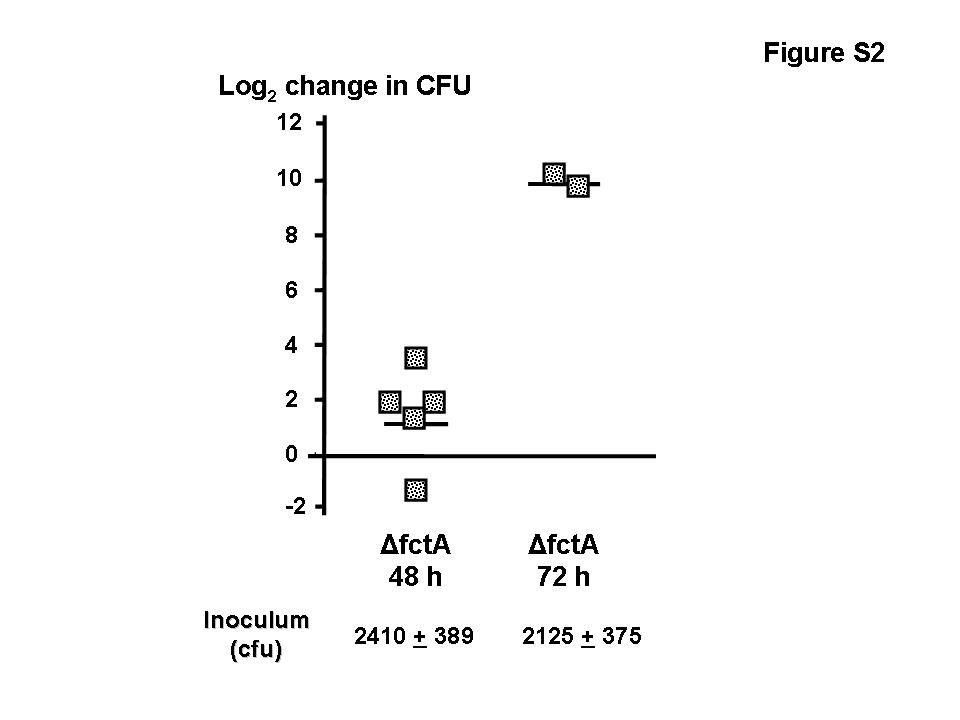

Supplement: Figure S2 — Absence of assembled pili has no effect growth rate at the skin during early stages of infection. The number of bacterial population doublings (log2 change in cfu) at the skin by 48 or 72 h post-inoculation with mid-logarithmic phase broth cultures of the Alab49 ΔfctA mutant (diamonds). Bars depict average mean values. The mean average inoculum dose and standard deviations are also indicated. (0.06 MB TIF) [file pone.0003450.s002.tif]
