# Supplementary material for: Impact of Orthologous Gene Replacement on the Circuitry Governing Pilus Gene Transcription in Streptococci
Source: PLoS One. 2008 Oct 20;3(10):e3450. doi: 10.1371/journal.pone.0003450 (PMC2565503; doi:10.1371/journal.pone.0003450)
Supplement: Table S1 — (0.08 MB PDF) [file pone.0003450.s003.pdf]

Table S1: Oligonucleotide primers used for gene inactivation and allele replacement.

description of target	nucleotide sequence (5' to 3') *
3' end of upstream flank	CTATATT***CCTAGG***TGAATGTAAATCAGCGTTAAAACAATAAACAACCTG
5' end of downstream flank	CTATTCTA***CTCGAG***ACAGTGCGGTGAGGGATTTCCACATCACCAATC
forward primer for aphA3	CGAGGTATGAAAACGAGAATTGGACCTTTACAG
reverse primer for aphA3	**TCCGGA**TCTAGGTACTAAAACAATTCATCCAG
fusion of 5' end of aph3A to the upstream flank	CTGTAAAGGTCCAATTCTCGTTTTCATACCTCGGTAATAGCTCTCTCCTAAACTTCTAGTCTAC
fusion of 3' end of aph3A to the downstream flank	CTGGATGAATTGTTTTAGTACCTAGA**TCCGGA**CTTATTAAGAGATGATATCTATCAAATTCCAAG
forward primer for aad9	ATCGATTTTCGTTCGTGAATACATGTTATAATAAC
reverse primer for aad9	GTAGAAG**TCCGGA**CGGTTATAATTTTTTTAATCTGTTATTTAAATAG
fusion of 5' end of aad9 to the 3' end of nra	CATGTATTCACGAACGAAAATCGATTACTTATCCAGTAACTGAGAAATGTAGAAG
fusion of 3' of aad9 to the downstream flank	CTATTTAAATAACAGATTAAAAAAATTATAACCG**TCCGGA**CAAACATTTTCCTTTATGCTATAATCTAAG
fusion of 5' end of aad9 to the 3' end of rofA	CATGTATTCACGAACGAAAATCGATGTATTGACAGGATTGCTTGAAAAATCAATACAG
fusion of 5' end of rofA promoter region to the upstream flank	CTCATATATGTTTTTGAGAGGAGAGAAAGAATTCACTATTAGAATAGCTATTCAGTG

* For the primers that link via fusion the aphA3 or aad9 genes to the flanking Alab49 regions, the sequence is underlined. Sequences in ***bold italics*** indicate restriction sites introduced for potential cloning purposes. A BspE1 site (in **bold**) was also introduced.
