# Supplementary material for: Impact of Orthologous Gene Replacement on the Circuitry Governing Pilus Gene Transcription in Streptococci
Source: PLoS One. 2008 Oct 20;3(10):e3450. doi: 10.1371/journal.pone.0003450 (PMC2565503; doi:10.1371/journal.pone.0003450)
Supplement: Table S2 — (0.08 MB PDF) [file pone.0003450.s004.pdf]

Table S2: Primers used for qRT-PCR.

Target gene	Forward primer	Reverse primer	Size of amplicon (bp)
cpa	AATATGCTGAAGGTGACTACTC	TATGTACCATCCGACAACTGC	138
emm53	AATAGAGCAGACGACGCTAG	TTCCCGTAAATCAGTCTTAAGC	132
fctA	TTGAACCAGATACTACTGCAAG	AGCAGACTCTTTATCTGTATTG	121
fctB	AGACAGCACTGTTCAAACTAGC	TCGTCTATTGTTTTCATCGCATC	113
hsp33	ATGGCACAGGTAACCCTTACACCTC	ACATTAAGTCCGATTGCAGATGGTG	117
mga2	CTTATCCAACATTCCTCAGAAATCC	CGTCGAGATGTAACCCAAATTTG	72
msmR	TTTGTCACTACATTATTGCCATTG	AGCTCTTCTTTGCTACGATGAG	117
nra	AACGTCATCACAACCTCTGA	TCTCAAGCCAAATTCCCGTAG	112
prtF2	AATATGCCTCTAAGTATACAAG	TTAGTTGGGTAACCTTCTGTC	96
recA	ATTGATTGATTCTGGTGCGG	ATTTACGCATGGCCTGACTC	139
sipA2	TGAGTCCTGCTTTAAGTGCAGGTG	AGCTTGAGCGACAATTCGACCAAC	127
ska	CGCAATGCCACATAAACTTG	AAGTAGACCTTGCCGTTTCG	130
speB	TGTCGGTAAAGTAGGCGGAC	GAGCTGAAGGGTTTAGTGCG	130
srtC2	GTTAGCTTTGAATGAGGATGTC	ATGGCAACACTGCCATCAACTG	125
up-3	GGTTGCAGGAAACTAATATTAG	CCCTCCTTCTAAACTAAAGTGGC	132
up-4	GAAACAGGCGAATGATTGACC	CAGTAATCCGATCGTCGTTTGTCGCCG	139
